# Immuno-modulatory activity of *Ganoderma lucidum*-derived polysacharide on human monocytoid dendritic cells pulsed with Der p 1 allergen

**DOI:** 10.1186/1471-2172-12-31

**Published:** 2011-05-25

**Authors:** Rong-Hwa Jan, Teng-Yi Lin, Ya-Chun Hsu, Shiuh-Sheng Lee, Shih-Yen Lo, Mingi Chang, Li-Kuang Chen, Yu-Li Lin

**Affiliations:** 1 Institute of Medical Sciences, Tzu-Chi University, Hualien, Taiwan; 2 Department of Pediatrics, Hualien Tzu-Chi Hospital, Hualien, Taiwan; 3 Department of Laboratory Medicine, Buddhist Tzu Chi General Hospital, Hualien, Taiwan; 4 Department of Biochemistry, National Yang-Ming University, Taipei, Taiwan; 5 Department of Medical Research, National Taiwan University Hospital, Taipei, Taiwan

**Keywords:** dendritic cells, Th1/Th2 cells, PS-G, asthma

## Abstract

**Background:**

*Ganoderma lucidum*-derived polysaccharide (PS-G) can rapidly and effectively promote the activation and maturation of immature dendritic cells (DCs), suggesting that PS-G possesses the capacity to regulate immune responses. This study aimed to clarify the immunologic effect of PS-G on monocyte-derived dendritic cells (MD-DCs) from asthmatic children allergic to house dust mites. The MD-DCs were stimulated for 24 h with the related allergen, Der p 1, in the presence or absence of PS-G. Cell surface markers and phagocytic capacity were assessed by FACS analysis, and key polarizing cytokines (IL-12 p40, IL-12 p70, IL-6, IL-23, and IL-10) were quantified. The subsequent regulatory effect of pulsed MD-DCs on naïve T cells was evaluated by determining the T-cell cytokine profile.

**Results:**

PS-G induced the maturation of MD-DCs and decreased phagocytic capacity, even if pulsed with Der p 1. After incubation with PS-G and Der p 1, MD-DCs produced higher amounts of IL-12 p70, IL-12 p40, IL-6, IL-23, and IL10 than Der p 1-pulsed DCs. Furthermore, type 1 helper T (Th1) cell cytokine (INF-γ) production was highly increased when naïve autologous T cells were co-cultured with Der p 1-pulsed MD-DCs. Naïve T cells stimulated by MD-DCs pulsed with Der p 1 failed to produce proliferation of T-cells, whereas the addition of PS-G to Der p 1 induced a significant proliferation of T-cells similar to that observed with PS-G alone.

**Conclusion:**

The presence of PS-G in an allergen pulse promoted allergic MD-DCs to produce IL-12 p70, IL-12 p40, IL-6, IL-23, and IL-10, and exerted an effect on shifting the immune balance towards Th1 in children with allergic asthma.

## Background

Allergic asthma is a common childhood disease that often persists into adulthood. The prevalence of childhood allergic diseases has increased dramatically in recent decades in many parts of the world [1], and children who mount an immune response to inhalant allergens have an increased risk of developing asthma. This immune response includes both IgE antibodies and type 2 helper T (Th2) cells, which are thought to contribute to inflammation in the respiratory tract. Moreover, sensitization to indoor allergens (dust mites, cats, and dogs) is strongly associated with asthma. The allergic disorders and diseases are characterized by predominant Th2 cytokine (IL-4, IL-5, and IL-13) production [2].

*Ganoderma lucidum*, a medicinal mushroom, is among the most popular herbal medicines in East Asia. *G. lucidum *has been reported to be effective in modulating immune functions, inhibiting tumor growth, and in the treatment of chronic hepatopathy, hypertension, neoplasia, and hyperglycemia [3-5]. *G. lucidum *has also been used to prevent and treat atopic diseases in several mouse and human models [6-8]. The main functional components of *G. lucidum *include polysaccharides, proteins, peptides, amino acids, and triterpenes. Polysaccharides are well-known for their immunomodulatory and anti-tumor functions [9] by reportedly enhancing the cytotoxic activity of natural killer cells and increasing tumor necrosis factor-α and interferon-γ release from macrophages and lymphocytes, respectively [10,11]. The polysaccharide component from *G. lucidum *(PS-G) has also been reported to elicit anti-apoptotic effects on neutrophils, which primarily depend on the activation of Akt-regulated signaling pathways [12]. We previously demonstrated that PS-G can rapidly and effectively promote the activation and maturation of immature healthy human dendritic cells (DCs), and promote T helper 1 immune responses in mice, thereby suggesting that PS-G may possess a potential capacity for regulating immune responses [13,14].

Dendritic cells are powerful antigen-presenting cells, the primary function which is to capture, process, and present antigens to naïve T cells [15,16]. Immature DCs reside in non-lymphoid tissues where DCs can capture and process antigens. Fully-mature DCs have a high surface expression of major histocompatibility complex (MHC) class II and co-stimulatory molecules (CD80 and CD86), but a decreased capacity to internalize antigens [17]. The induction of DC maturation is critical for the induction of Ag-specific T lymphocyte responses and may be essential for the development of human vaccines relying on T cell immunity. IL-12 production is also an important marker for DC maturation and can be used to select Th1-inducing adjuvants. IL-10, a cytokine that inhibits inflammatory and cell-mediated immune responses [18], has enormous potential for treating inflammatory and autoimmune disorders. IL-23, which is mainly produced by macrophages and DCs [19], was recently identified as a cytokine that induces IL-17 expression [20]. IL-17 production is enhanced in acute atopic dermatitis lesions [21] and allergic contact dermatitis [22].

The purpose of the current study was to determine the potential immune modifications that PS-G directly affects at the level of DCs in children with allergic asthma. The development of a defined T-cell profile is highly dependent on DC-stimulating factors, and the direct effect of PS-G on allergic MD-DCs may underline its potential and natural regulatory activity by directing the development of allergic reactions.

## Results

### Der p 1-pulsed MD-DCs increased maturation in the presence of PS-G

**Figure 1:**
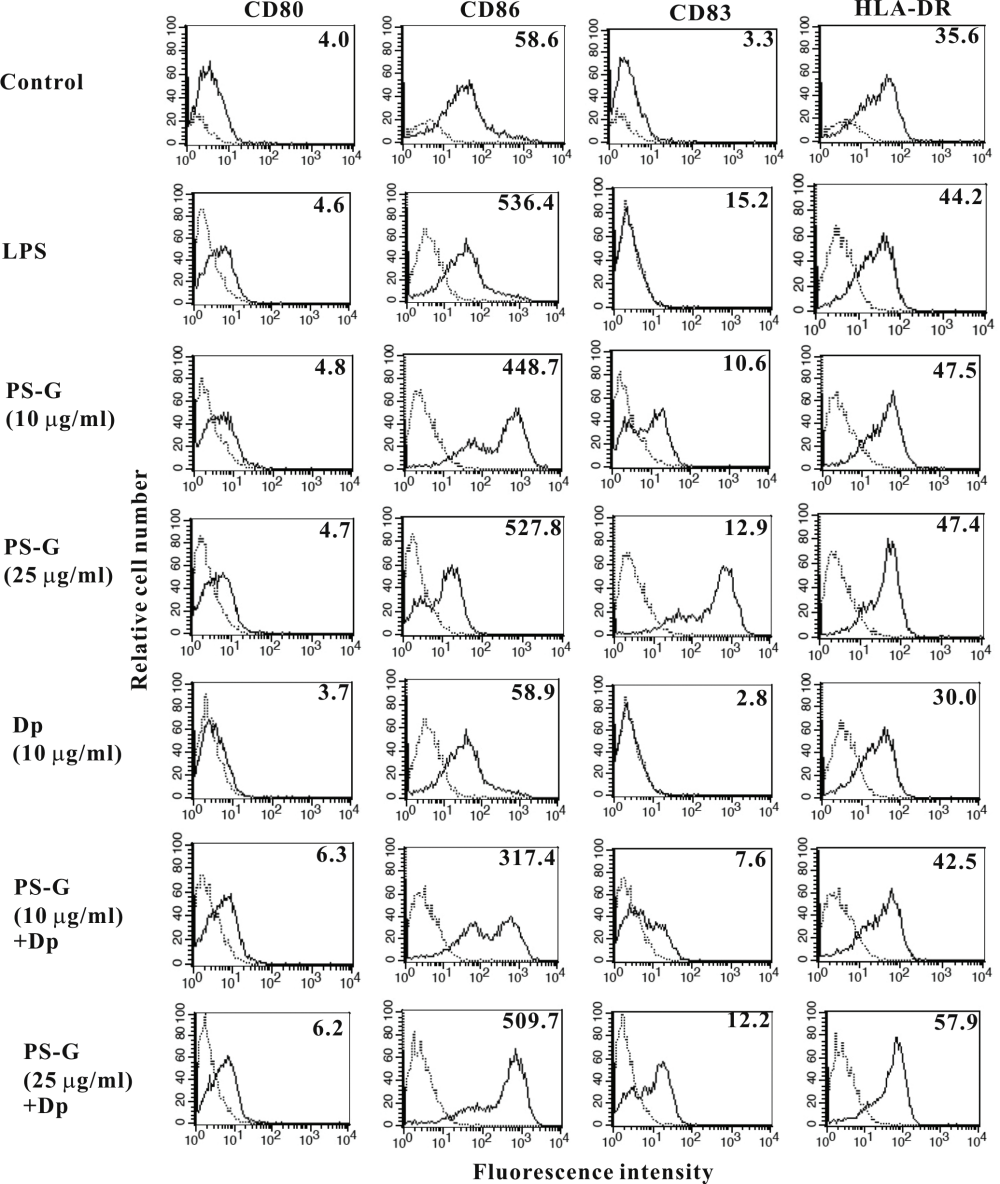
**PS-G increased co-stimulatory molecule expression on MD-DCs**. Immature MD-DCs were pulsed either with Der p 1 (1 μg/ml) or PS-G (10 or 25 μg/ml), or both for 24 h and analyzed by flow cytometry for CD80, CD86, CD83, and HLA-DR expression. DCs stimulated with LPS (100 ng/ml) were positive maturation controls. Values shown are the mean fluorescence intensity (MFI) indexes. Dotted line, isotype control; solid line, specific mAbs. One representative of three independent experiments is shown.

MD-DCs displayed a mature cell phenotype similar to that of LPS-stimulated DCs in the presence of PS-G, and was characterized by an increase in CD80, CD86, CD83, and HLA-DR expression (Figure 1) and a decrease in the CD1a marker (data not shown). When PS-G was added to Der p 1-stimulated MD-DCs, the phenotypic profile was similar to that detected in DCs stimulated with PS-G alone.

### PS-G decreased the endocytotic ability of mature MD-DCs

**Figure 2:**
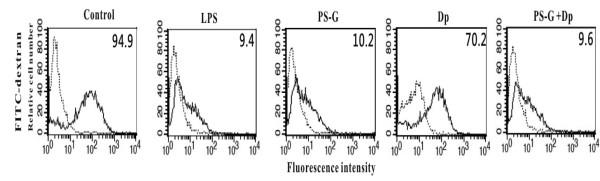
**The effect of PS-G on the phagocytic capacity of MD-DCs**. Immature MD-DCs (1 × 10^6 ^cells/1 ml per well) were incubated for 24 h either with Der p 1 (1 μg/ml) or PS-G (10 or 25 μg/ml) or both. DCs stimulated with LPS (100 ng/mL) were positive maturation controls. Cells were then incubated with FITC-dextran for 1 h at 4°C (dotted lines) or 37°C (solid lines). Values shown in the flow cytometry profiles are the MFI indexes. One representative of three independent experiments is shown.

Immature DCs that capture and process antigens (through phagocytosis, macro-pinocytosis, and adsorptive endocytosis) lose the ability to take up antigen and become mature DCs [15]. The uptake of FITC-dextran has been shown to be maximal in immature human MD-DCs through macro-pinocytosis and the mannose receptor [13]. To determine whether or not PS-G modulates the antigen uptake ability of MD-DCs, FITC-dextran was analyzed by flow cytometry (Figure 2). LPS and PS-G-treated MD-DCs showed decreased FITC-dextran uptake compared to untreated control MD-DCs and MD-DCs treated with Der p 1 alone. However, PS-G altered the phagocytic capacity of MD-DCs with Der p 1 alone.

### PS-G increased IL-12 p70, IL-12 p40, Il-6, IL-23, and IL-10 production by DCs with Der p 1 allergen

**Figure 3:**
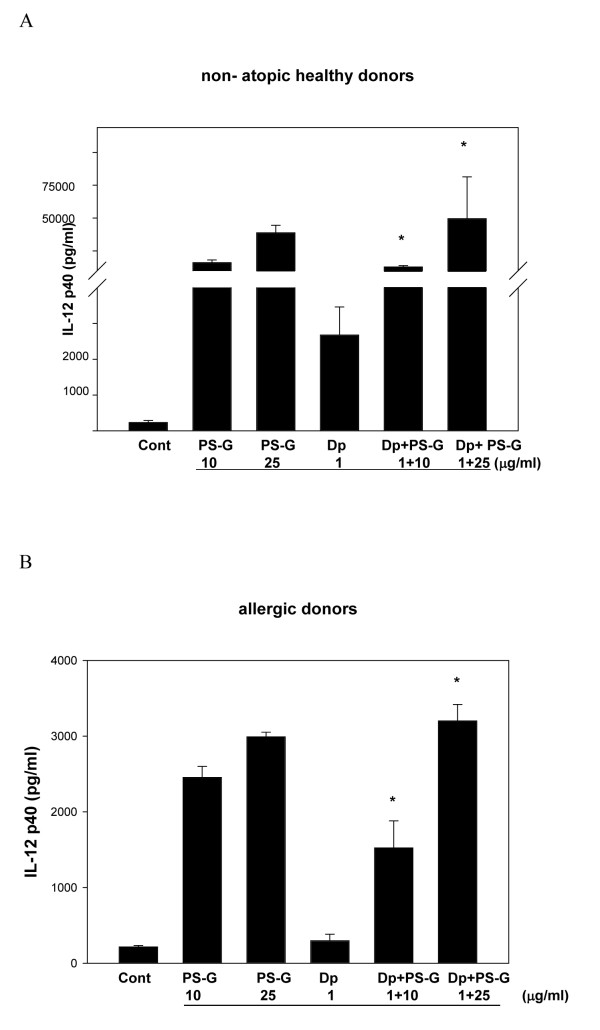
**PS-G increased IL-12 p40 production in Der p 1-pulsed MD-DCs from five healthy children (A) and six children with allergic asthma (B)**. MD-DCs were incubated for 24 h with Der p 1, PS-G, or both. The supernatants were collected for cytokines. Data are represented as the mean ± SE for three determinations. Statistical analysis focused on DCs with or without PS-G in the presence of Der p 1. *p < 0.05. (Cont = control)

**Figure 4:**
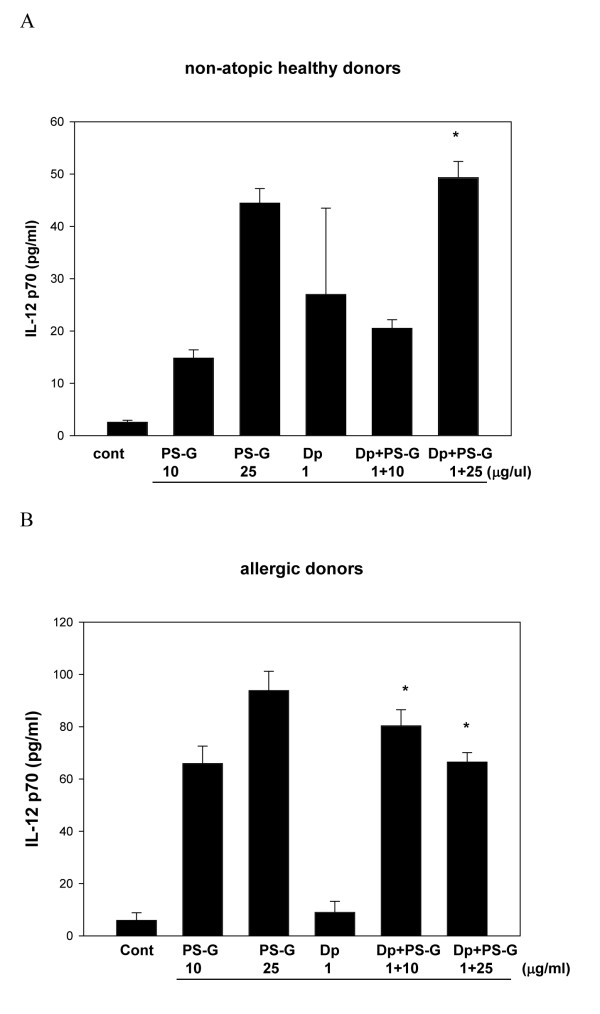
**PS-G increased IL-12 p70 production in Der p 1-pulsed MD-DCs from five healthy children (A) and six children with allergic asthma (B)**. MD-DCs were incubated for 24 h with Der p 1, PS-G, or both. The supernatants were collected for cytokines. Data are represented as the mean ± SE for three determinations. Statistical analysis focused on DCs with or without PS-G in the presence of Der p 1. *p < 0.05. (Cont = control)

**Figure 5:**
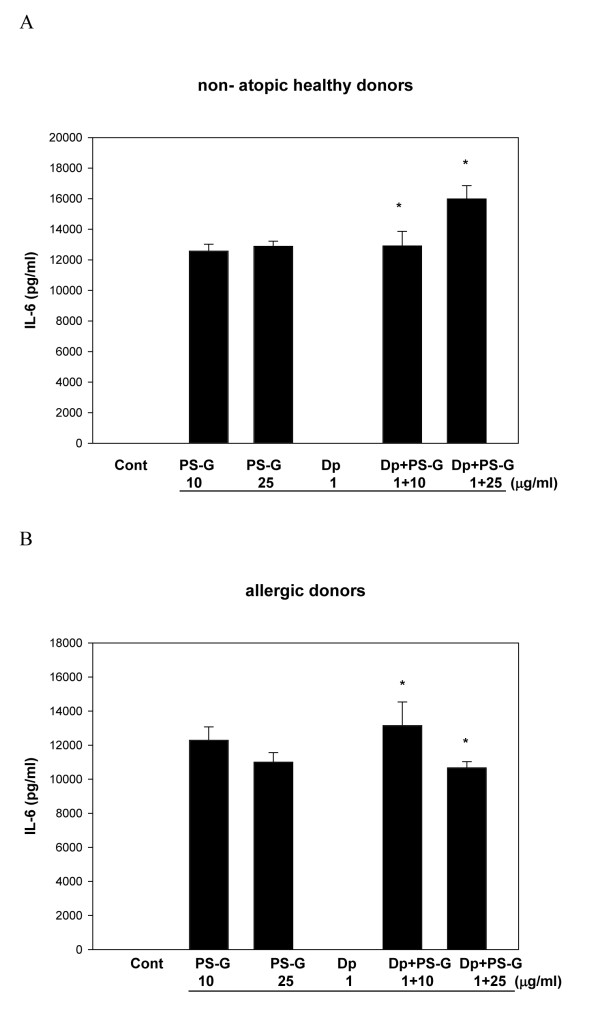
**PS-G increased IL-6 production in Der p 1-pulsed MD-DCs from five healthy children (A) and six children with allergic asthma (B)**. MD-DCs were incubated for 24 h with Der p 1, PS-G, or both. The supernatants were collected for cytokines. Data are represented as the mean ± SE for three determinations. Statistical analysis focused on DCs with or without PS-G in the presence of Der p 1. *p < 0.05. (Cont = control)

**Figure 6:**
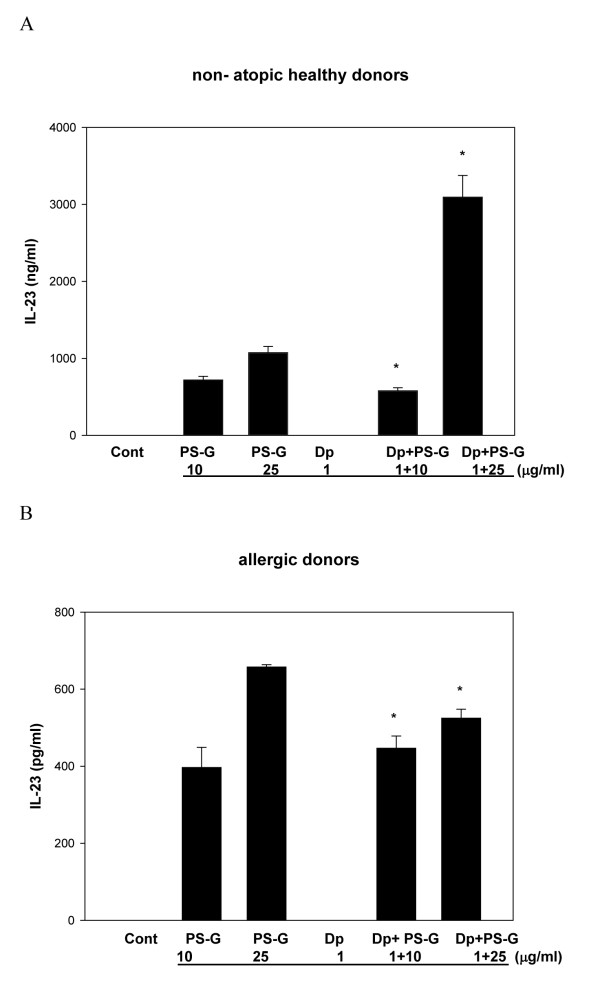
**PS-G increased IL-23 production in Der p 1-pulsed MD-DCs from five healthy children (A) and six children with allergic asthma (B)**. MD-DCs were incubated for 24 h with Der p 1, PS-G, or both. The supernatants were collected for cytokines. Data are represented as the mean ± SE for three determinations. Statistical analysis focused on DCs with or without PS-G in the presence of Der p 1. *p < 0.05. (Cont = control)

**Figure 7:**
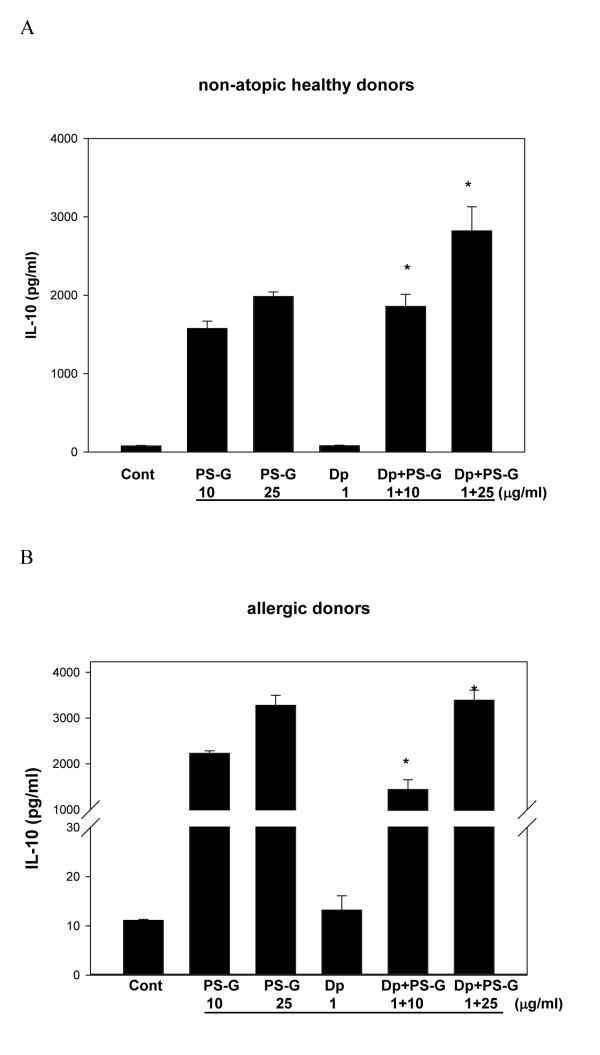
**PS-G increased IL-10 production in Der p 1-pulsed MD-DCs from five healthy children (A) and six children with allergic asthma (B)**. MD-DCs were incubated for 24 h with Der p 1, PS-G, or both. The supernatants were collected for cytokines. Data are represented as the mean ± SE for three determinations. Statistical analysis focused on DCs with or without PS-G in the presence of Der p 1. *p < 0.05. (Cont = control)

Because IL-12, IL-10, IL-6, and IL-23 are known to play key roles in the modulation of T cell responses [23], IL-12 p70, IL-12 p40, IL-6, IL-23, and IL-10 produced by MD-DCs pulsed only with Der p 1, PS-G, or both were evaluated. Der p 1-pulsed DCs failed to secrete the production of IL-12 p70, IL-12 p40, IL-6, IL-23, and IL-10 in allergic asthmatic children (Figures 3B, 4B, 5B, 6B, 7B), whereas the reactivity of DCs to Der p 1 was very weak in allergic patients. In healthy donors, Der p 1-pulsed DCs produced large amounts of IL-12 p40 and IL-12 p70 (Figures 3A and 4A).

Interestingly, PS-G increased IL-12 p70 and IL-12 p40 production in Der p 1-pulsed DCs from allergic patients, while IL-12 p40 levels remained similar to the IL-12 p40 levels obtained in the absence of Der p 1. Moreover, PS-G induced dose-dependent IL-10 production, which remained at a similar level in the presence of Der p 1. PS-G also increased IL-6 and IL-23 production in MD-DCs pulsed with Der p 1 or PS-G alone. Based on IL-12 p70, IL-6, and IL-10 production, DC reactivity towards PS-G appeared to be equal in allergic patients and healthy donors, but there were high levels of IL-12 p40 in healthy donors. Thus, PS-G was shown to induce DCs from allergic patients to produce the pro-Th1 cytokine IL-12, as observed in healthy donors.

### PS-G decreased T_H_2 cytokine production by CD4-naive T cells

The polarization of the primary autologous T cells induced by MD-DCs was then examined. To evaluate the influence of PS-G on T-cell polarization, Der p 1-pulsed MD-DCs incubated with or without PS-G were further co-cultured with autogeneic naïve CD4^+ ^T cells. The production of Th1 cytokines (IFN-γ), Th17 cytokines (IL-17A), and Th2 cytokines (IL-4, IL13, and IL-5) were analyzed.

**Figure 8:**
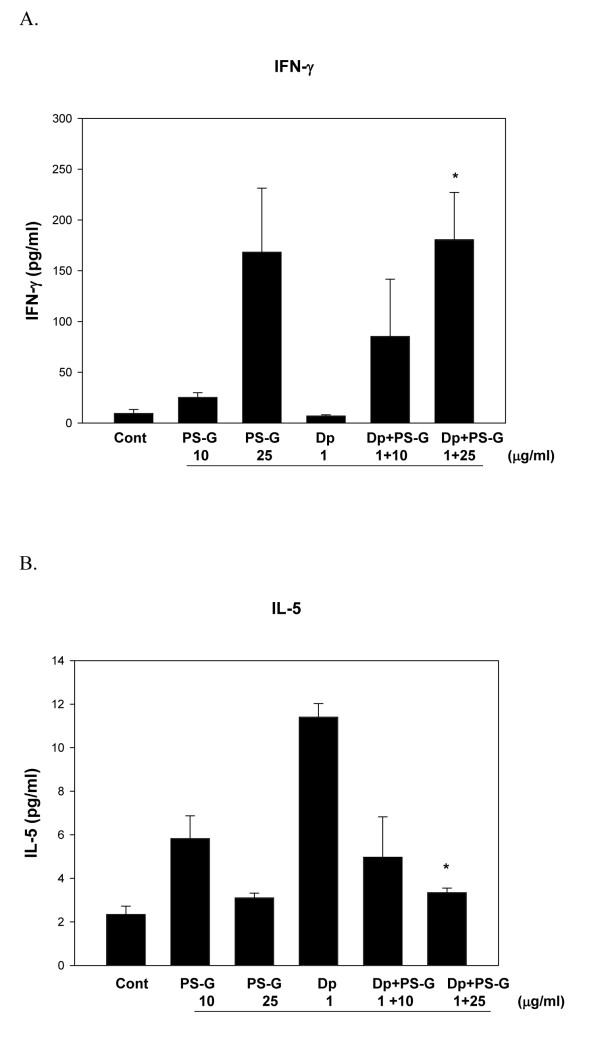
**PS-G affected T cell cytokine response**. MD-DCs from five children with allergic asthma were pulsed with Der p, PS-G, or both for 24 h and cultured with autogenic naïve T cell. Supernatants were analyzed for IFN-g (A) and IL5 (B), which were produced by activated T cells after 2 days of culture. Data are represented as means ± SEM of triplicates and representative of three independent experiments. Statistical analysis focused on DCs with or without PS-G in the presence of Der p 1. *p < 0.05. (Cont = control)

Stimulation of naïve T cells from allergic patients with DCs pulsed only with PS-G, not only Der p 1, induced high IFN-γ production. The addition of PS-G to the Der p stimulus significantly modified IFN-γ secretion (Figure 8A). When autologous naïve CD4^+^T cells from allergic asthmatic children were incubated with Der p 1-pulsed DCs, production of IL-5 was observed; however, this production was significantly decreased in Der p 1-pulsed DCs with PS-G (Figure 8B). The production of IL-4, IL-13, and IL-17A were not significantly changed (data not shown).

### PS-G induced T-cell proliferation by Der p 1-pulsed MD-DCs

**Figure 9:**
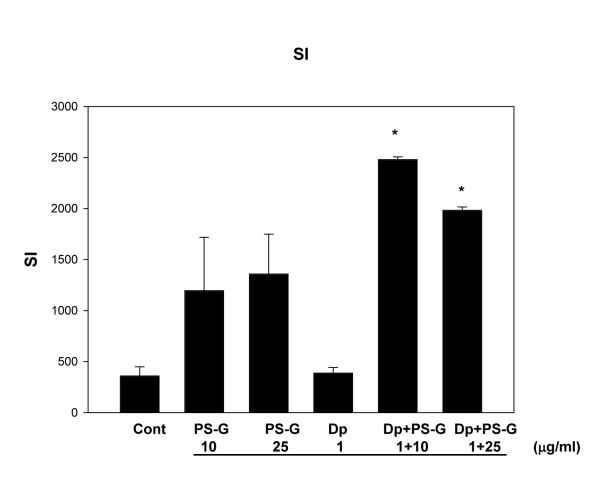
**PS-G affected T cell proliferation response**. MD-DCs from five children with allergic asthma were pulsed with Der p, PS-G, or both for 24 h and cultured with autogenic naïve T cell. Autologous T-cell proliferation was measured after 3 days of co-culture with MD-DC. The stimulation index (SI) was calculated as a mean counts per minute (cpm) of stimulated wells divided by the mean cpm of control wells. Values are presented as the mean stimulation index for triplicate wells. Data are represented as the mean ± SE of five determinations. Statistical analysis focused on DCs with or without PS-G in the presence of Der p 1. *p < 0.05. (Cont = control)

When naïve CD4^+ ^T cells were co-cultured for 3 days with Der p 1-pulsed MD-DCs from children with allergic asthma, the proliferation of T cells was low compared to unpulsed DCs (Figure 9). The addition of PS-G to Der p 1 induced a significant proliferation of T-cells similar to that observed with PS-G alone.

## Discussion

*Ganoderma lucidum *(G. lucidum: Lingzhi in Chinese, Reishi in Japanese) has been used for a long time in China and other Asian countries to prevent and treat various human diseases. *Ganoderma lucidum *has been the subject of modern pharmacologic and clinical research in the last 30 years, and is reportedly effective in promoting health and longevity, and enhancing the efficacy of treatment of many diseases. The main aim of this study was to determine the regulatory effect of PS-G on MD-DCs from children with allergic asthma. The results showed that PS-G increased the expression of CD80, CD86, CD83, and HLA-DR molecules on the cell membranes of Der p 1-pulsed MD-DCs. Even though the maturation markers of MD-DCs from children with allergic asthma treated with PS-G alone increased, the effect was as clear as the effect in MD-DCs treated with LPS alone. Taken together, PS-G or LPS alone induces the maturation of MD-DCs from allergic asthma patients, and LPS- and PS-G-treated MD-DCs showed decreased antigen processing compared to control MD-DCs and MD-DCs treated with Der p 1 alone.

We also evaluated the production of cytokines (IL-12 p40, IL-12 p70, IL-6, IL-23, and IL-10) in MD-DCs from allergic asthma children, and found that PS-G alone stimulated Der p 1-pulsed DCs to secrete IL-12 p40, IL-12 p70, IL6, IL23, and IL-10. The different responses of PS-G on DCs from healthy and allergic donors are particularly noteworthy. PS-G induced a large amount of IL-12 p40 from healthy compared to atopic donors. IL-12 is a heterodimeric cytokine produced by activated macrophages, neutrophils, and dendritic cells. Endogenous IL-12p40 selectively inhibits AHR and airway remodeling in an asthma model with prolonged antigen exposure [24]. IL-12B is located in this genomic region and encodes IL-12p40 [25]; however, the allelic effects of this polymorphism on asthma susceptibility and asthma-related phenotypes has been shown. The first report suggested that the heterozygous genotype was more frequently detected in children with severe asthma [26]. The genetic variation in the promoter of IL-12B displays functional activity and cytokine production capacity.

We also showed that the amount of IL-23 produced by PS-G on DCs from healthy donors was higher than allergic donors (Figure 3D). The p40 subunit of IL-12 is shared by IL-23, a cytokine with actions similar to, but distinct from IL-12 [27]. A protective role for the Th1 response generated by IL-12/IL-23 has been suggested based on infectious diseases in children with genetic defects of the IL-12/23 - IFN-γ circuit [28].

PS-G-induced IL-12 p40 and IL-12 p70 secretion, and shifted the immune balance towards Th1. The data presented also shows that PS-G induces IL-10 secretion. Recent observations indicate that DCs play an important roles in activating regulatory T (Treg) cells, including induction of CD4^+ ^T cells into Foxp3^+ ^Treg cells *in vitro *[29], IL-10-producing CD4+ Treg cells [30], and regulating the balance of Th1/Th2 immunity [31].

Cytokines are involved in orchestrating the initiation and maintenance of allergic inflammatory responses. Th2 cytokines, such as IL-4, IL-5, and IL-13, are involved in the development and maintenance of the allergic immune response. The Th1 cytokine, IFN-γ, is known to down-regulate Th2 responses by antagonizing IL-4. The selective production of IFN-γ is tightly regulated and is highly dependent on the reactivity of DCs to the environment, which in turn contributes to the T-cell polarization process. PS-G increases IL-6 and IL-23 production in DCs, although IL-6 and IL-23 appear to play a role in the induction of IL-17 by human CD4+ T cells, IL-1β, and TGF-β, in combination with other factors, which are essential in the induction of IL-17 production [23]. Thus, the production of IL-17A by the stimulation of naïve T cells with DCs pulsed with PS-G, was not significantly changed in this study.

Th17 cytokines have been shown to have an association with allergic disease. Th17 cells are highly involved in the development and maintenance of psoriasis [32], and a possible role for IL-17, IL-22, and Th17 cells in atopic dermatitis or allergic contact dermatitis is emerging. Nickel-specific Th0, Th1, or Th2 clones from allergic contact dermatitis patients have been shown to produce IL-17 [33]. Elevated IL-17 concentrations have also been found in lung and blood of allergic asthma patients and has been linked to the severity of asthma [34,35]. Whether or not a specific role exists for Th17 cells in asthma, however, is controversial [36].

This is the first study to demonstrate that PS-G induces the production of Th1 cytokines produced by naïve human T cells through a direct effect on DCs in a model of children with allergic asthma. These results extend our previous findings that PS-G can induce gene expression changes in human DCs, and specifically promote Th1 cytokine release in BALB/c mice [14]. Other studies have reported on the immunomodulatory and adjuvant activities of PS-G in mice [37,38].

The cytokine environment encountered by a naïve CD4^+^T cell plays a prominent role in determining whether or not naïve CD4^+ ^T cells develop into Th1 or Th2 cells. Thus, the same naïve CD4^+^T cells can give rise to Th1 or Th2 cells under the influence of environmental (e.g., cytokine) and genetic factors. IFN-γ production is related to an increase in IL-12 p40 and IL-12 p70 production observed when MD-DCs from children with allergic asthma are pulsed by Der p 1 in the presence of PS-G. Taken together, we suggest DCs may be the key cell type in children with allergic asthma.

IL-12 also has an important function in promoting Th1 immune responses and limiting the establishment and maintenance of Th2-type responses, mainly by enhancing IFN-γ production and by providing an effective deviation signal during the early differentiation of Th0 cells [39]. Thus, IL-12 p40 and IL-12 p70 production by PS-G may contribute to switching the balance from an established Th2 response to a more pronounced Th1 response.

Attempts to evaluate the effect of PS-G on specific T-cell proliferation in allergic asthmatic children has led to an apparent dichotomy for naïve T cells. Whereas naïve T cells exhibit a weak proliferative response to Der p 1-pulsed DCs, increased T cell proliferation is observed with PS-G-treated MD-DCs. T-cell proliferation requires at least two signals, one through contact of antigen-MHC with T cell receptors and the other through an interaction between co-stimulatory molecules [40,41]. Thus, the varying degrees of DC maturation may explain the differences observed in T-cell proliferation. Similarly, lactic acid bacteria have been shown to have roles in modulating DC maturation from allergic patients [42].

Indeed, in response to Der p 1, DCs express quite low levels of co-stimulatory molecules compared to PS-G-pulsed DCs, which express intense up-regulation of the surface markers (i.e., CD80, CD83, CD86, and HLA-DR). There is an apparent relationship between the emergence of the PS-G-dependent T cell population and the increase in IFN-γ production.

## Conclusion

The results suggest that PS-G may switch the established Th2 response in allergic patients towards a long-lasting Th1 response, and may therefore represent a new therapeutic strategy for the treatment of children with allergic asthma.

## Methods

### Patients

Blood was collected from allergic patients sensitive to *Dermatophagoides pteronyssinus *and from non-atopic healthy children. All allergic children had a history of asthma and presented with the usual features of house dust mite sensitization, as follows: specific IgE antibodies (CAP class >3); and positive skin prick test responses to *D. pteronyssinus*. The total IgE level was >250 kU/L. The Research Ethics Committee of Hualien Tzu-Chi General Hospital approved the study and all subjects provided informed consent.

### PS-G purification from *G. lucidum*

As we previously reported [4], fruiting bodies of *G. lucidum *were washed, disintegrated, and extracted with boiling water for 8-12 h. The hot water extract was fractionated into a polysaccharide fraction (alcohol insoluble) and non-polysaccharide fraction (alcohol soluble). The crude polysaccharide obtained was then passed through a gel-filtration Sephadex G 50 column (Pharmacia, Uppsala, Sweden) and further purified by anion exchange chromatography with diethylaminoethyl-cellulose [3].

The polysaccharide from *Ganoderma lucidum *(PS-G) is a branched (1→6)-β-D-glucan moiety. To rule out possible endotoxin lipopolysaccharide (LPS) contamination, the LPS content was determined by the chromogenic Limulus Amebocyte Lysate assay, which showed no detectable levels of endotoxin (<0.10 endotoxin units/ml) in the PS-G samples.

### Human DC generation

The DCs were generated from peripheral mononuclear cells (PBMCs), as described previously [43,44], but with some modifications. Briefly, PBMCs were obtained from donors by Ficoll-Hypaque centrifugation (PharmaciaSweden). The light density fraction from the 42.5%-50% interface was recovered. CD14^+ ^cells were purified by positive selection using anti-CD14^+ ^micro-beads in conjunction with a MiniMACS system, following the manufacturer's instructions (Miltenyi Biotech, Auborn, CA, USA). The DC14^+ ^cells were cultured at 1 × 10^6 ^cells/ml of cRPMI in 24-well plates (Costar) with GM-CSF (800 U/ml) and IL-4 (500 U/ml). Fresh medium containing GM-CSF and IL-4 was added every 2-3 days. Human monocyte-derived DCs were routinely used on day 6 of culture.

### Activation of DCs

The MD-DCs (1 × 10^6 ^cells/1 ml per well) were incubated for 24 h with Der p 1 (1 μg/ml) or PS-G (10 or 25 μg/ml) or both. As a positive maturation control, DCs were stimulated with LPS (100 ng/ml). *Escherichia coli *LPS (L8274) was purchased from Sigma-Aldrich Chemical Co. (St. Louis, MO, USA).

### Flow cytometric analysis for surface markers

The DCs were harvested and washed with cold buffer (PBS containing 2% FCS and 0.1% sodium azide), incubated in cold buffer, and subsequently stained with FITC- or PE-labeled mAbs (anti-human CD86, CD80, CD83, HLA-DR or relevant isotype controls; BD Pharmingen) (Becton Dickinson, San Jose, CA) based on the manufacturer's instructions. The DC surface marker expression was analyzed using the CellQuest program, which excluded dead cells.

### Phagocytic capacity analysis

The MD-DCs were washed twice, re-suspended in 1 ml of RPMI 1640 medium containing 10% FCS, and placed on ice for 30 min. The cells were then incubated with FITC-labeled dextran (0.2 mg/ml; Invitrogen, Carlsbad, CA, USA) at 4°C or 37°C for 1 h. Finally, the cells were thrice-washed with cold buffer and analyzed with a FACSort cell analyzer.

### Autologous mixed leukocyte reaction (MLR)

The PBMCs were obtained as described above and naïve CD4^+^T cells were purified from PBMCs using magnetic beads (Miltenyi Biotec). The autologous naïve CD4^+^T cells were distributed at 1 × 10^6 ^cells per well and incubated for 5 days in the presence of 1 × 10^5 ^stimulated MD-DCs. Tritiated thymidine (1 μCi/well; New England Nuclear, Boston, MA, USA) incorporation for 16 h was determined using a liquid scintillation counter.

### Cytokine assay

#### DC activation

After a 24-h stimulation in the 4 conditions (non-pulsed or pulsed with Der p 1, PS-G, or Der p 1 plus PS-G), supernatants from the MD-DCs (1 × 10^6^) were collected, centrifuged, and tested via ELISA (R&D Systems, Minneapolis, MN, USA) for the presence of IL-6, IL-23, IL-10, IL-12 p70, and IL-12 p40.

#### T cell-DC co-cultures

After washing, 1 × 10^5 ^stimulated MD-DCs were incubated with 1 × 10^6 ^naïve CD4^+^T cells for 2 days. The co-cultures were performed in 48-well, flat-bottomed culture plates (1 ml/well). The presence of IL-13, IL-17A, IL-4, IL-5, and IFN-γ in the supernatants was detected by means of ELISA (R&D Systems).

### Statistical analysis

Parametric statistical analysis was performed with the Student's test, and a *p *< 0.05 was considered statistically significant.

## Abbreviations

PS-G: *Ganoderma lucidum*; DCs: dendritic cells; Th1: helper T type 1; IFN-γ: interferon-γ; LPS: lipopolysaccharides

## Authors' contributions

RWJ and YCH performed the experimental work, data analyses, interpretation of data, drafted the manuscript, and were involved in the conception and design of the study.

LKY and YLL were involved with data analyses, interpretation of data, drafted the manuscript, and made substantial contributions to the design of the study.

TYL, SSL, SY L, and MC made substantial contributions to the conception and design of the study. All authors have read and approved the final manuscript.
